# A Case of “Anaphylaxis” to NovoSeven in a Hemophiliac Patient

**DOI:** 10.7759/cureus.40802

**Published:** 2023-06-22

**Authors:** Andy Wang, Chisom Okezue, Lillian Chang, Yaakov Spira, Christopher Nabors

**Affiliations:** 1 Internal Medicine, Westchester Medical Center, Valhalla, USA

**Keywords:** paradoxical vocal fold motion, tranexamic acid, novoseven, hematoma, factor ix inhibitor, hemophilia b, wheezing, stridor, anaphylaxis

## Abstract

Anaphylaxis is a life-threatening emergency that may be confused with other less serious conditions. The onset of true anaphylaxis typically occurs within minutes following exposure to an offending agent, and it can variably include dyspnea/wheezing, hemodynamic compromise, rash, hives/pruritus, swelling, or gastrointestinal symptoms. The absence of an expected association between exposure(s) and classic symptoms should lead to the consideration of alternative diagnoses. Here, we describe the course of a patient with hemophilia B who developed stridor and wheezing after exposure to the recombinant factor VII, NovoSeven, and tranexamic acid (TXA) for the management of hematomas. Due to a reported prior history of anaphylaxis to multiple factor replacements, the patient’s initial management included NovoSeven with steroid/antihistamine prophylaxes and close monitoring with epinephrine at the bedside. Despite the administration of prophylaxis, the patient developed significant stridor, was treated with epinephrine and nebulizers and additional steroids, and was transferred to the intensive care unit. There, a pattern of NovoSeven administration followed variably by wheezing and stridor continued for two days until the patient’s respiratory condition was predictable and stable. The patient’s subsequent clinical course following transfer to the general medical ward was not consistent with anaphylaxis. This case highlights the importance of evaluating for mimickers of anaphylaxis, especially where only select symptoms such as stridor and wheezing are present without other serious signs of anaphylaxis such as hypoxemia, hypotension, or significant tachycardia.

## Introduction

Anaphylaxis is an acute life-threatening hypersensitivity reaction that can present with hemodynamic instability and respiratory distress including stridor, wheezing, and hypoxia [1,2]. In the United States, the lifetime prevalence of anaphylaxis has been reported to be approximately 0.05%-2% [3]. Anaphylaxis can be difficult to diagnose when the clinical presentation does not include a close temporal association between exposure and symptom onset and/or where only select symptoms are present [1,4]. For instance, paradoxical vocal fold motion (PVFM), laryngospasm, and foreign body obstruction can similarly present with stridor, while reactive airway processes can present with severe dyspnea and wheezing [1,4-6]. Here, we present a case of a patient with hemophilia B who developed stridor and wheezing after the administration of the recombinant factor VII, NovoSeven. He was initially treated for presumed anaphylaxis. However, during a 10-day hospitalization, he continued to have recurrent stridor and wheezing without other signs of hemodynamic or respiratory compromise. The symptoms did not recur consistently in close temporal relation to NovoSeven administration and were not necessarily improved with steroids/antihistamines and epinephrine. Further, the symptoms persisted following a switch from NovoSeven to tranexamic acid (TXA). This pattern suggested that the patient was not having true anaphylaxis.

## Case presentation

**Figure 1:**
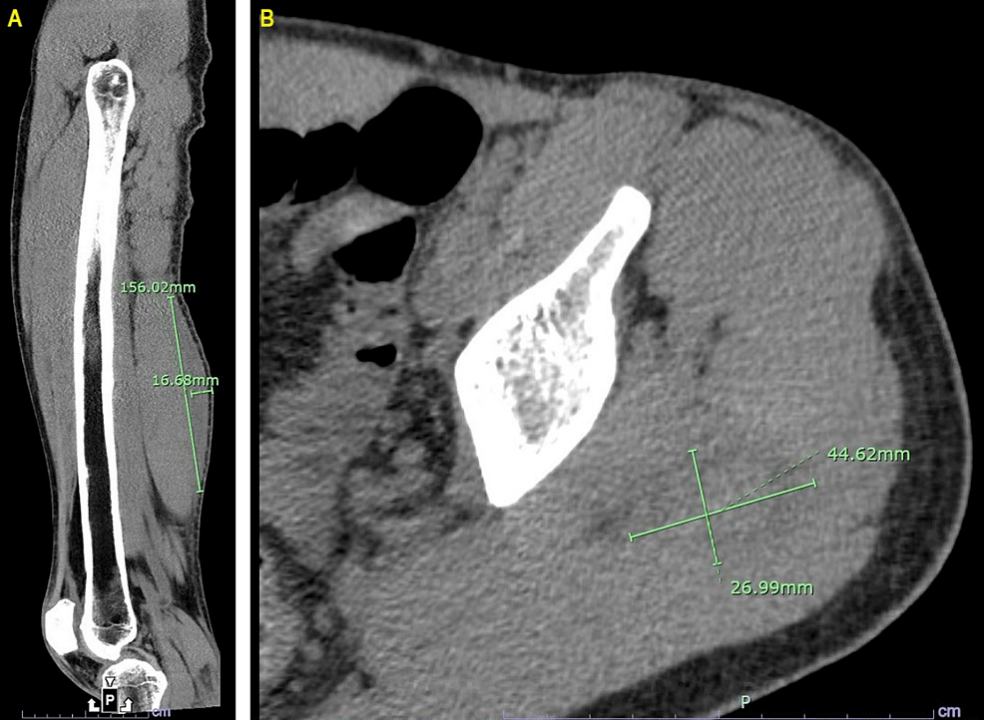
Computed tomography scan Computed tomography scan showing hematomas in the left posterior thigh musculature (A) and gluteus maximus (B).

A 33-year-old male (an inmate at the time of presentation, but no longer incarcerated) with severe hemophilia B, factor IX inhibitors, minor falls leading to subdural hemorrhage, and upper and lower extremity hematomas requiring fasciotomy for compartment syndrome presented to the hospital with left posterior thigh and buttock pain after suffering a minor fall from his bed. Vital signs were as follows: heart rate of 78 beats per minute, blood pressure of 102/80 mmHg, respiratory rate of 16 breaths per minute, and oxygen saturation of 99% on room air. On examination, the patient was noted to have left flank pain with tenderness to palpation and swelling in the left gluteal region as well as over the parietal aspect of his skull. Laboratory findings were notable for a prothrombin time of 14.7 seconds (normal range: 9.4-12.5 seconds), international normalized ratio of 1.3 (normal range: 0.90-1.10), activated partial thromboplastin time (aPTT) of 91.2 seconds (normal range: 25.0-36.5 seconds), aPTT mixing study of 34.4 seconds (normal range: 12.0-36.5 seconds), and factor IX activity level of 1% (normal range: 85%-190%), consistent with moderate hemophilia B. Computed tomography demonstrated hematomas in the left posterior thigh musculature and gluteus maximus and small biparietal scalp hematomas without intracranial bleed (Figure 1A, 1B).

On admission, the patient provided (and available records reflected) a history of anaphylaxis to factor IX therapy, the presence of factor IX inhibitors, and multiple episodes of “anaphylaxis” (throat tightness, stridor, wheezing, and hives) following NovoSeven administration(s) three years earlier, which led to multiple hospitalizations. The patient reported no history of asthma, chronic obstructive pulmonary disease (COPD), or reactive airway disease. Despite premedication with intravenous (IV) diphenhydramine during this hospitalization, the patient experienced stridor initially 12 hours after the administration of NovoSeven, leading to a rapid response call. At that time, the patient reported pruritus, dyspnea, and tongue and lip swelling. Vital signs were as follows: heart rate of 88 beats per minute, blood pressure of 132/87 mmHg, respiratory rate of 22 breaths per minute, and oxygen saturation of 99% on room air. On physical examination, the patient had marked stridor, but no accessory muscle use, tongue or lip swelling, angioedema, or urticaria. He was administered IV methylprednisolone, IV diphenhydramine, racemic epinephrine, and nebulizers with resolution of symptoms over 2-3 hours. Given a need for further NovoSeven administration, the patient was transferred to the intensive care unit for close monitoring. There he received NovoSeven doses every four hours after premedication with hydrocortisone and diphenhydramine, as well as administration of nebulizers and epinephrine, as needed. Despite this regimen, the patient experienced repeated episodes of stridor and/or wheezing without respiratory decompensation. The time-to-onset of stridor and wheezing episodes varied greatly, ranging from seconds to hours following NovoSeven administration.

As the patient’s hematomas stabilized/improved over the next 36 hours, NovoSeven was discontinued, TXA was started, and he was transferred to a general ward location, where he stayed for seven days. During this time, the patient developed similar episodes of stridor and/or wheezing without hypoxemia or hemodynamic instability after TXA use. The absence of classical features of anaphylaxis led to the consideration of a psychological component to the respiratory symptoms, so the psychiatry service was consulted. Their team observed the patient to have stridor and wheezing independent of TXA administration that appeared at times to be exaggerated and improved or even resolved during a conversation. On the other hand, one attending physician from the primary team noted the onset of wheezing during a routine examination where the patient was initially comfortable and in good spirits but which was followed within half an hour by an episode of stridor. Of note, the patient endorsed many life stressors and anxiety preceding and during his incarceration, which could have been an underlying contributor to a psychogenic component of the patient’s condition. The patient was therefore offered therapy and/or a trial of psychiatric medications, which he declined. Otolaryngology was also consulted and performed flexible laryngoscopy, which did not reveal an anatomical anomaly, PVFM, or other cause of stridor. Over an eight-day course, the patient’s premedication with corticosteroids was tapered without worsening of stridor/wheezing. Given the stability of his hematomas, improved coagulation parameters, and very low suspicion for anaphylaxis to NovoSeven or TXA, the patient was discharged after a total of 10 days and instructed to self-administer NovoSeven along with diphenhydramine premedication, the only medication the patient believed consistently improved his symptoms. He was recommended to undergo further outpatient workup with pulmonary function testing (PFT) and consultation with an allergist/immunologist.

## Discussion

Anaphylaxis can be difficult to diagnose when there is suspicion of other conditions that can mimic its presentation [1,4]. As such, it is important to understand how anaphylaxis typically presents and to keep open the possibility of alternate diagnoses. Anaphylaxis is an acute severe systemic reaction that can affect multiple organ systems, including the heart, lungs, gastrointestinal tract, and skin [1,2,4]. It can present with a variety of symptoms including pruritus, cough, throat tightness, dyspnea, vomiting, abdominal pain, diarrhea, dizziness, and syncope [1]. Vital signs may demonstrate hemodynamic instability including hypotension, tachycardia, and hypoxia [2]. Physical examination findings can include urticaria, angioedema, flushing, stridor, wheezing, and cyanosis [1]. Anaphylaxis generally occurs in close temporal proximity (minutes to an hour or two) to a trigger such as a medication or food allergen [1]. In the present case, the patient’s history of “anaphylaxis” coupled with severe stridor and wheezing, and apparent anxiety/physical discomfort following NovoSeven administration led to initial treatment for presumed anaphylaxis. However, despite pre- and post-medication with anaphylaxis therapy and switching from NovoSeven to TXA, the patient continued to have episodes of stridor and wheezing that varied in severity, duration, and temporal relation to NovoSeven or TXA exposure but did not have other clinical signs of anaphylaxis including hypotension, hypoxia, tachycardia, hives, or angioedema.

Cases of recombinant factor VII-induced anaphylaxis have been reported, although their presentations differ from the current case. One case reported a patient who experienced acute severe systemic vasodilation within 30 seconds of recombinant factor VII administration complicated by cardiac arrest during thoracic aortic surgery, with circulatory recovery within three minutes of epinephrine administration and cardiac massage [7]. Another case reported a patient who developed tachycardia, periorbital edema, and confusion within 20 minutes of receiving recombinant factor VII, with resolution of symptoms after administration of epinephrine [8].

Although the current patient’s clinical course did not support a diagnosis of anaphylaxis, it is unclear what led to his recurrent episodes of stridor and wheezing. Many conditions can mimic the presentation of anaphylaxis and present with stridor, including foreign body obstruction, malignancy, and PVFM (also known as vocal cord dysfunction) (Table 1) [5]. Of these entities, PVFM was considered a strong possibility in this case as stridor was the most prominent and consistent symptom. In symptomatic episodes of PVFM, the vocal cords adduct during inspiration or expiration, leading to stridor, a sensation of throat tightness, and cough [4]. The gold standard for diagnosis is laryngoscopy during a symptomatic episode, which can show abnormal vocal cord motion [4]. Treatment of PVFM typically involves reassurance, breathing maneuvers (i.e., breathing through a straw or nose and diaphragmatic breathing), and administration of a helium and oxygen mixture (heliox) in refractory cases [4]. As our patient was asymptomatic during laryngoscopy, further evaluation with PFT and repeat laryngoscopy during a symptomatic episode would be needed to confirm or exclude PVFM.

**Table 1:** Some causes of stridor in adults This table lists some acute and chronic etiologies of stridor in adults [1,4,5].

Some etiologies of stridor in adults			
Acute	Supportive findings	Chronic	Supportive findings
Anaphylaxis/allergy	Urticaria, pruritus, mucosal swelling/orofacial edema, flushing, wheezing	Neoplasm/compressive mass	History of heavy smoking or alcohol use, weight loss, night sweats, worsening stridor with increasing size of mass
Post-extubation laryngeal edema	History of recent endotracheal intubation, respiratory distress	Vocal cord paralysis and dysfunction	History of recent surgery or intubation, may be accompanied by neurological findings (i.e., muscle weakness)
Vocal cord dysfunction	Recurrent, episodic, choking sensation, throat tightness, cough		
Foreign body obstruction	Abrupt onset, respiratory distress, wheezing, cough		
Angioedema	Orofacial swelling including lips and tongue, possible history of hereditary angioedema or angiotensin-converting enzyme inhibitor use		

The stridor characteristic of PVFM can occasionally be mistaken for wheezing and sometimes lead to a misdiagnosis of asthma [4]. Further, patients can also have concurrent PVFM and asthma, making it challenging to differentiate between the two [4]. One study reported that 59% of patients with PVFM had a prior diagnosis of asthma [4]. In the present case, given the patient had intermittent episodes of isolated wheezing, it is possible the patient may have had underlying asthma, COPD, or reactive airway disease, although the patient denied a supportive history and his wheezing did not respond fully to treatment with nebulizers, hydrocortisone, epinephrine, and diphenhydramine. Outpatient PFT would be needed to further investigate the underlying etiology and could support a diagnosis of PVFM in the presence of characteristic flattening of the inspiratory limb of the flow-volume loop [4].

Studies have demonstrated a link between PVFM and psychiatric conditions including anxiety and post-traumatic stress disorder (PTSD) [4]. Wareing and Mitchell [9] reported a case of a patient who presented with intermittent inspiratory stridor that only manifested when the patient’s mother was present, with laryngoscopy consistent with PVFM, which was felt to be due to family stressors. They also reported another case of a patient with inspiratory stridor but a normal voice and no oxygen desaturation [9]. Upper airway endoscopic examination was normal, and the patient later revealed that she had been experiencing stressors at home [9]. They also reported a third case of a patient with two weeks of intermittent inspiratory stridor refractory to inhaler therapy [9]. That patient had a normal otolaryngological examination but reported significant stress due to issues with schooling arrangements [9]. The patient was diagnosed with psychogenic stridor, which resolved after her schooling arrangements were addressed [9]. In the present case, it is possible that there was a psychosomatic component to the patient’s presentation, given his status as a prisoner, reported anxiety, and the potential for PTSD related to a host of life stressors. This was supported by the observation that his stridor/wheezing was exaggerated during psychiatric evaluation and improved during a distracting conversation. Further, it is possible that the patient achieved secondary gain from his condition, which removed him from prison to a more pleasant hospital environment. While there remains uncertainty regarding the basis for the patient’s symptoms, his clinical course indicated that his condition, while appearing quite serious, did not reflect a life-threatening set of anaphylactic reactions. Despite multiple prior similar hospital admissions, the patient continued to carry a diagnosis of anaphylaxis to coagulation factors/therapies, which potentially led to multiple courses of therapy and extended hospitalizations that could potentially have been avoided.

## Conclusions

This case highlights the importance of evaluating for mimickers of anaphylaxis. Alternative underlying etiologies should be considered in the presence of only select symptoms such as stridor and wheezing without other serious signs of anaphylaxis as well as in the absence of a clear temporal relation between exposure and symptom onset. Early recognition of a clinical pattern not consistent with anaphylaxis holds the potential to spare patients potentially unnecessary therapies and intensive care unit monitoring.
